# Lung Disease and Unequal Pupils

**Published:** 1908-06-06

**Authors:** 


### LUNG DISEASE AND UNEQUAL PUPILS.

Slight inequality of the pupils, temporary or permanent, is so common, even in health, that it may almost be regarded as a normal abnormality. Inequality sufficient to attract immediate attention is less common, though even that condition is occasionally observed in perfectly healthy people. It is of interest, therefore, to note that the definite inequality of the pupils to which attention is often attracted in cases of lung disease is not a point from which any definite conclusions can be drawn. It may be said that this is an entirely negative point, and so it is; but negative points are often valuable things to know, for it is very unsatisfactory to be confronted with a well-marked clinical condition, and yet to be in doubt as to whether it "means anything" or not.

It has more than once been taught that a dilated pupil upon the same side as the abnormal lung signs is an indication of empyema as distinct from serous effusion. It becomes quite clear, however, from the statistics recorded by Massolongo, Signorelli, and others that the inequality of the pupils, though common enough in lung cases, is so inconstant in its behaviour that nothing definite can be deduced from the sign.

Taking all pleuro-pulmonary cases together, decided inequality of the pupils may be noted in about one-third. The incidence in different lung conditions may be briefly summarised as follows:—

| | Per cent. of cases having unequal pupils. |
|---|---|
| Tuberculosis } Acute pneumonia } ... | 40 |
| Pleuritic effusion ... | 25 |
| Acute bronchitis ... | 20 |
| Emphysema ... | 12 |
| Chronic bronchitis ... | 10 |

Where the lung disease is unilateral there seems to be no special tendency for the pupillary abnormality to be upon that side; nor do the pupillary symptoms throw any light upon the nature or source of the pulmonary disease, though upon the whole the more acute the lesion the more likely are the pupils to be unequal. The performance of paracentesis thoracis in cases of pleural effusion does not at once do away with the pupil differences, so that the latter do not even help in the difficult distinction between persistence of compressed lung on the one hand and re-accumulation of fluid upon the other. Sometimes the pupil upon the same side as the lung trouble is persistently smaller than the other; sometimes it is persistently larger; sometimes, again, one pupil is the smaller of the two one day, whereas it is the other way round the next.

